# The Susceptibility and Potential Functions of the LBX1 Gene in Adolescent Idiopathic Scoliosis

**DOI:** 10.3389/fgene.2020.614984

**Published:** 2021-01-18

**Authors:** Ming Luo, Yuxiao Zhang, Shishu Huang, Yueming Song

**Affiliations:** ^1^Department of Orthopedics, Orthopedic Research Institute, West China Hospital, Sichuan University, Chengdu, China; ^2^West China Hospital and West China School of Medicine, Sichuan University, Chengdu, China

**Keywords:** adolescent idiopathic scoliosis, LBX1, genome-wide association study, susceptibility, curve progression, etiology

## Abstract

Genome-wide association studies have identified many susceptibility genes for adolescent idiopathic scoliosis (AIS). However, most of the results are hard to be replicated in multi-ethnic populations. LBX1 is the most promising candidate gene in the etiology of AIS. We aimed to appraise the literature for the association of LBX1 gene polymorphisms with susceptibility and curve progression in AIS. We also reviewed the function of the LBX1 gene in muscle progenitor cell migration and neuronal determination processes. Three susceptibility loci (rs11190870, rs625039, and rs11598564) near the LBX1 gene, as well as another susceptibility locus (rs678741), related to LBX1 regulation, have been successfully verified to have robust associations with AIS in multi-ethnic populations. The LBX1 gene plays an essential role in regulating the migration and proliferation of muscle precursor cells, and it is known to play a role in neuronal determination processes, especially for the fate of somatosensory relay neurons. The LBX1 gene is the most promising candidate gene in AIS susceptibility due to its position and possible functions in muscle progenitor cell migration and neuronal determination processes. The causality between susceptibility loci related to the LBX1 gene and the pathogenesis of AIS deserves to be explored with further integrated genome-wide and epigenome-wide association studies.

## Introduction

Adolescent idiopathic scoliosis (AIS) is the most common pediatric spinal deformity, defined as a lateral spinal curvature with a Cobb angle of >10 degrees, and it affects millions of children worldwide (Cheng et al., 2015). In contrast to congenital, neuromuscular, and syndromic scoliosis, an agreed-upon theory of the pathogenesis of AIS is lacking. The increased risk of developing AIS in first-degree relatives and higher AIS concordance rates in monozygotic twins than in dizygotic twins provide strong evidence for the heritability of AIS (Yee et al., 2014; Simony et al., 2016). Genome-wide association studies (GWASs) have identified many susceptibility loci as well as candidate genes such as ER1, PAX1, LBX1, GPR126, and SLC39A8 (Takahashi et al., 2011; Kou et al., 2013; Zhu et al., 2015; Haller et al., 2018). However, replication studies for most susceptibility loci are hard to verify these associations in multi-ethnic populations.

In the region containing the LBX1 gene (encoding ladybird homeobox 1), three susceptibility loci, rs11190870, rs625039, and rs11598564 at chromosome 10q24.31 were identified in Japanese AIS patients (Takahashi et al., 2011). A functional variant rs678741 encoding an antisense transcript of LBX1 was also reported to be associated with AIS (Zhu et al., 2015). Recently, the association between LBX1 gene polymorphisms and AIS was identified in Asian and Caucasian populations with a meta-analysis (Jiang et al., 2019). The LBX1 gene specifies distinct neuronal subtypes in the spinal cord and hindbrain, and it is also essential for limb muscle development in vertebrates (Jagla et al., 1995; Hernandez-Miranda et al., 2018). Coincidentally, dysfunctional somatosensory and asymmetric paraspinal muscle were proposed to explain the pathogenesis of AIS (Cheng et al., 2015). Therefore, LBX1 is a promising candidate gene involved in the etiology of AIS.

In this narrative review, we aimed to appraise the literature for the association of LBX1 gene polymorphisms with AIS susceptibility and curve progression. Besides, we reviewed the function of the LBX1 gene in muscle progenitor cell migration and neuronal determination processes. Moreover, integrated genome-wide and epigenome-wide association studies for the LBX1 gene in AIS are prospected. This review provides new insights into the potential role of the LBX1 gene in the etiology of AIS.

## The Association of Variants Near LBX1 With AIS Susceptibility

GWASs have identified many susceptibility SNPs as well as promising candidate genes for AIS. Except for the LBX gene, most of the results of these GWASs failed to be replicated in different ancestral populations. Takahashi et al. (2011) first identified three common variants near LBX1 in Japanese AIS, and rs11190870, which lies 7.5 kb downstream of the LBX1 gene, was the most significant SNP (*P* = 1.24 × 10–19). Subsequently, the susceptibility locus was successfully replicated in 10 other studies, including six studies with a Chinese population (Fan et al., 2012; Gao et al., 2013; Jiang et al., 2013; Zhu et al., 2015; Nada et al., 2018; Man et al., 2019), one study with a Caucasian population (Chettier et al., 2015), one study with a Scandinavian population (Grauers et al., 2015), one study with a French-Canadian population (Nada et al., 2018), and one study with a Japanese population (Kou et al., 2019). Notably, the association between locus rs11190870 and AIS was still the most significant in the recent GWAS with 79,211 Japanese individuals included (*P* = 2.01 × 10–82) (Kou et al., 2019).

A consistent result was obtained in six meta-analyses for the susceptibility locus of rs11190870. Londono et al. (2014) performed a meta-analysis of the locus of rs11190870 with multiple ethnic groups, and the results firmly established the LBX1 region as the first major susceptibility locus for AIS. Two other meta-analyses conducted in East Asian populations also confirmed this susceptibility locus in AIS, and the T allele of rs11190870 was considered the risk allele (Chen et al., 2014; Liang et al., 2014). Recently, two updated meta-analyses identified the robust significant association of rs11190870 in Asian and Caucasian AIS patients (Cao et al., 2016; Jiang et al., 2019). The detail information for the association of rs11190870 with AIS susceptibility are shown in Table 1.

**Table 1:** The association of rs11190870 with AIS susceptibility.

**Study design**	**References**	**Population**	**No. AIS/Control**	**RAF AIS/Control**	***P*-value**	**OR 95% CI**
GWAS	Takahashi et al., 2011	Japanese	1,376/11,297	0.670/0.565	1.24 × 10^−19^	1.56 (1.41–1.71)
Case–control	Fan et al., 2012	Southern Chinese	300/788	0.67/0.52	9.1 × 10^−10^	1.85 (1.52–2.25)
Case–control	Gao et al., 2013	Han Chinese	513/440	0.620/0.490	1.17 × 10^−8^	1.70 (1.42–2.04)
Case–control	Jiang et al., 2013	Han Chinese	949/976	0.597/0.496	1.8 × 10^−9^	1.51 (1.33–1.71)
GWAS	Zhu et al., 2015	Chinese	4,317/6,016	0.63/0.52	8.68 × 10^−14^	1.56 (1.39–1.75)
GWAS	Chettier et al., 2015	Caucasian	620/1,287	0.651/0.551	5.43 × 10^−9^	1.52 (1.32–1.75)
Case–control	Grauers et al., 2015	Scandinavian	1,739/1,812	0.69/0.36	7.0 × 10^−18^	1.53 (1.39–1.69)
Case–control	Liu et al., 2017	Northern Chinese	180/180	0.58/0.46	1.34 × 10^−3^	1.62 (1.20–2.17)
GWAS	Nada et al., 2018	French-Canadian	788/952	0.63/0.58	4.68 × 10^−3^	1.22 (1.06–1.39)
GWAS	Kou et al., 2019	Japanese	5,327/73,884	0.66/0.56	2.01 × 10^−82^	1.52 (1.46–1.59)
Case–control	Man et al., 2019	Chinese	313/200	0.612/0.502	5.0 × 10^−4^	1.56 (1.21–2.01)
Meta-analysis	Londono et al., 2014	Multiple ethnic	5,159/17,840	-	1.22 × 10^−43^	1.6 (1.5–1.7)
Meta-analysis	Chen et al., 2014	East Asian	3,215/15,347	-	<0.001	1.61 (1.51–1.72)
Meta-analysis	Liang et al., 2014	East Asian	2,889/5,526	-	<0.001	1.61 (1.50–1.72)
Meta-analysis	Cao et al., 2016	Multiple ethnic	5,754/18,628	-	<0.001	1.21 (1.17–1.25)
Meta-analysis	Jiang et al., 2019	Multiple ethnic	-	-	<0.00001	1.54 (1.48–1.61)

In Takahashi's study, the other two susceptibility loci near LBX1, the rs625039 and rs11598564 polymorphisms, were also associated with AIS (Takahashi et al., 2011). The rs625039 SNP was located in the 5′-flanking region of the LBX1 gene, and two case–control studies successfully replicated the susceptibility locus in the Southern and Northern Chinese Han populations (Gao et al., 2013; Liu et al., 2017). In addition, two meta-analyses conducted in East Asian populations also confirmed this susceptibility locus in AIS, and the G allele of rs625039 was considered the risk allele (Cao et al., 2016; Jiang et al., 2019). The rs11598564 SNP was located in the 3′-flanking region of the LBX1 gene, and it was also repeatable in the Chinese Han population (Gao et al., 2013; Liu et al., 2017). Two meta-analyses conducted in East Asian populations also confirmed that the G allele of rs625039 was the risk allele in AIS (Cao et al., 2016; Jiang et al., 2019). The detail information for the association of rs625039 and rs11598564 with AIS susceptibility are shown in Table 2.

**Table 2:** The association of rs625039, rs11598564, and rs678741 with AIS susceptibility.

**SNP**	**Study design**	**References**	**Population**	**No. AIS/Control**	**RAF AIS/Control**	***P*-value**	**OR 95% CI**
rs625039	GWAS	Takahashi et al., 2011	Japanese	1,376/11,297	0.724/0.636	8.13 × 10^−15^	1.49 (1.34–1.64)
	Case–control	Gao et al., 2013	Han Chinese	513/440	0.714/0.626	5.09 × 10^−5^	1.49 (1.23–1.80)
	Case–control	Liu et al., 2017	Northern Chinese	180/180	0.66/0.57	2.45 × 10^−2^	1.41 (1.04–1.90)
	Meta-analysis	Cao et al., 2016	Multiple ethnic	1,646/13,749	-	<0.001	1.14 (1.11–1.17)
	Meta-analysis	Jiang et al., 2019	Multiple ethnic	-	-	<0.00001	1.50 (1.38–1.62)
rs11598564	GWAS	Takahashi et al., 2011	Japanese	1,376/11,297	0.542/ 0.460	5.98 × 10^−14^	1.42 (1.30–1.56)
	Case–control	Gao et al., 2013	Han Chinese	513/440	0.600/0.497	5.54 × 10^−6^	1.52 (1.27–1.83)
	GWAS	Zhu et al., 2015	Chinese	4,317/6,016	0.67/0.60	2.15 × 10^−8^	1.33 (1.19–1.52)
	Meta-analysis	Cao et al., 2016	Multiple ethnic	1,966/13,585	-	<0.001	1.21 (1.16–1.25)
	Meta-analysis	Jiang et al., 2019	Multiple ethnic	-	-	<0.0001	1.41 (1.31–1.51)
rs678741 (LBXAS1)	GWAS	Zhu et al., 2015	Chinese	4,317/6,016	0.543/0.451	9.68 × 10^−37^	1.44 (1.37–1.52)
	GWAS	Chettier et al., 2015	Caucasian	620/1,287	0.627/0.527	5.56 × 10^−9^	1.52 (1.32–1.72)
	GWAS	Nada et al., 2018	French-Canadian	788/952	0.55/0.58	0.112	1.11 (0.97–1.28)
	Case–control	Man et al., 2019	Chinese	319/201	0.575/0.447	<0.0001	1.67 (1.30–2.15)
	Meta-analysis	Cao et al., 2016	Multiple ethnic	4,937/7303	-	<0.001	1.20 (1.18–1.23)
	Meta-analysis	Jiang et al., 2019	Multiple ethnic	-	-	<0.0001	1.35 (1.16–1.59)

In the first GWAS to investigate susceptibility loci in Chinese AIS, the rs678741 polymorphism, located in the intron of the LBX1AS1 gene, had the strongest association with AIS susceptibility (*P* = 9.68 × 10–37) (Zhu et al., 2015). The findings for this susceptibility locus were replicated in Caucasian AIS with a strong association (Chettier et al., 2015). In a GWAS of the French-Canadian population, the SNP rs678741 showed a significant association with severe AIS (Nada et al., 2018). Recently, the association with disease onset for rs678741 was successfully replicated in the Chinese population (Man et al., 2019). Two meta-analyses conducted in multi-ethnic populations also confirmed this susceptibility locus in AIS, and the A allele of rs678741 was considered the risk allele (Cao et al., 2016; Jiang et al., 2019).

## The Association of Variants Near LBX1 With AIS Severity

Although AIS affects millions of children with a global pooled prevalence of 1.34%, the prevalence of curvature exceeding the surgical threshold is much lower (Lonstein, 2006; Cheng et al., 2015). The ability to distinguish individuals at high risk of curve progression would facilitate early treatment, which is believed to be efficient in patients and economic for their families (Weinstein et al., 2013; Agabegi et al., 2015). An AIS prognostic test, called “ScoliScores,” was proposed to predict progression in the Caucasian population according to 53 related SNPs (Ogilvie, 2010; Ward et al., 2010). Regrettably, the ScoliScores were not replicable in different ethnic populations (Ogura et al., 2013; Roye et al., 2015; Tang et al., 2015; Xu et al., 2016).

As the most promising candidate gene in AIS susceptibility, the value of the LBX1 gene in predictions of AIS curve progression is still unclear. Jiang et al. (2013) first investigated the association of rs11190870 with curve progression of AIS in a Han Chinese population, and patients with the TT genotype had a larger Cobb angle (*P* = 0.005). Subsequently, four studies, including two with a Chinese population and two with a Japanese population, could not replicate the association of rs11190870 with the curve severity of AIS (Gao et al., 2013; Takahashi et al., 2015, 2018; Man et al., 2019). The detail information for the association of rs11190870 with AIS severity are shown in Table 3.

**Table 3:** The association of rs11190870, rs11598564, rs625039, and rs678741 with AIS severity.

**SNP**	**References**	**Population**	**No. AIS**	**Cobb angle of TT**	**Cobb angle of TC**	**Cobb angle of CC**	***P*-value**
rs11190870	Jiang et al., 2013	Chinese	314	34.1 ± 11.6	32.0 ± 13.8	27.2 ± 9.4	**0.005**
	Gao et al., 2013	Chinese	234	30.10 ± 14.81	30.73 ± 19.56	27.90 ± 18.05	0.33
	Takahashi et al., 2015	Japanese	2,068	39.0 ± 15.4	40.2 ± 16.4	37.6 ± 14.6	0.20
	Takahashi et al., 2015	Japanese	123	43.6 ± 16.4	37.1 ± 14.0	30.6 ± 13.1	0.07
	Takahashi et al., 2018	Japanese	1,860	41.7 ± 16.5	42.0 ± 16.6	39.3 ± 15.5	0.13
	Man et al., 2019	Chinese	176	47.2 ± 15.3	47.4 ± 19.4	45.0 ± 16.9	0.679
**SNP**	**References**	**Population**	**No. AIS**	**Cobb angle of AA**	**Cobb angle of AG**	**Cobb angle of GG**	***P*****-value**
rs625039	Gao et al., 2013	Chinese	234	31.35 ± 22.07	28.73 ± 17.89	31.00 ± 16.68	0.37
**SNP**	**References**	**Population**	**No. AIS**	**Cobb angle of AA**	**Cobb angle of AG**	**Cobb angle of GG**	***P*****-value**
rs11598564	Gao et al., 2013	Chinese	234	28.32 ± 17.26	30.60 ± 19.58	30.18 ± 15.02	0.50
**SNP**	**References**	**Population**	**No. AIS**	**Cobb angle of GG**	**Cobb angle of GA**	**Cobb angle of AA**	***P*****-value**
rs678741 LBX1AS1	Zhu et al., 2015	Chinese	632	38.1 ± 8.2	36.3 ± 9.1	37.9 ± 8.4	0.29
	Man et al., 2019	Chinese	175	46.6 ± 16.9	48.7 ± 18.4	44.8 ± 17.4	0.412

The other two variants near LBX1, rs11598564 and rs625039, were also explored in the Chinese population, but no association was found between the susceptibility loci and the severity of curvature (Gao et al., 2013). The novel locus rs678741, which is near the region encoding an antisense transcript of the LBX1 gene, was associated with AIS susceptibility but not with curve severity in the Chinese population (Zhu et al., 2015) Recently, a study on the association of rs678741 with AIS curve progression also failed to replicate the association in a Chinese population (Man et al., 2019). The detail information for the association of rs11598564, rs625039, and rs678741 with AIS severity are shown in Table 3.

The LBX1 gene is essential for neuron and muscle development, and LBX1 is the most promising candidate gene in AIS susceptibility because of its position and possible function. The associations of three susceptibility loci near LBX1 (rs11190870 and rs11598564, located in the 3′-flanking regions, and rs625039, located in the 5′-flanking region) were successfully verified in multi-ethnic populations. In addition, another susceptibility locus, rs678741, which has the potential to regulate the expression of the LBX1 gene, was also identified to have a robust significant association in multi-ethnic AIS patients. However, the association of the four variants related to LBX1 with AIS severity failed to be replicated. These results suggested that the LBX1 gene might be involved in the initiation but not in the progression of AIS. Because susceptibility does not mean a functional variant, the functions of the LBX1 gene and its potential roles in the pathogenesis of AIS need to be clarified.

## The Function of the LBX1 Gene in Muscle Development

**Figure 1:**
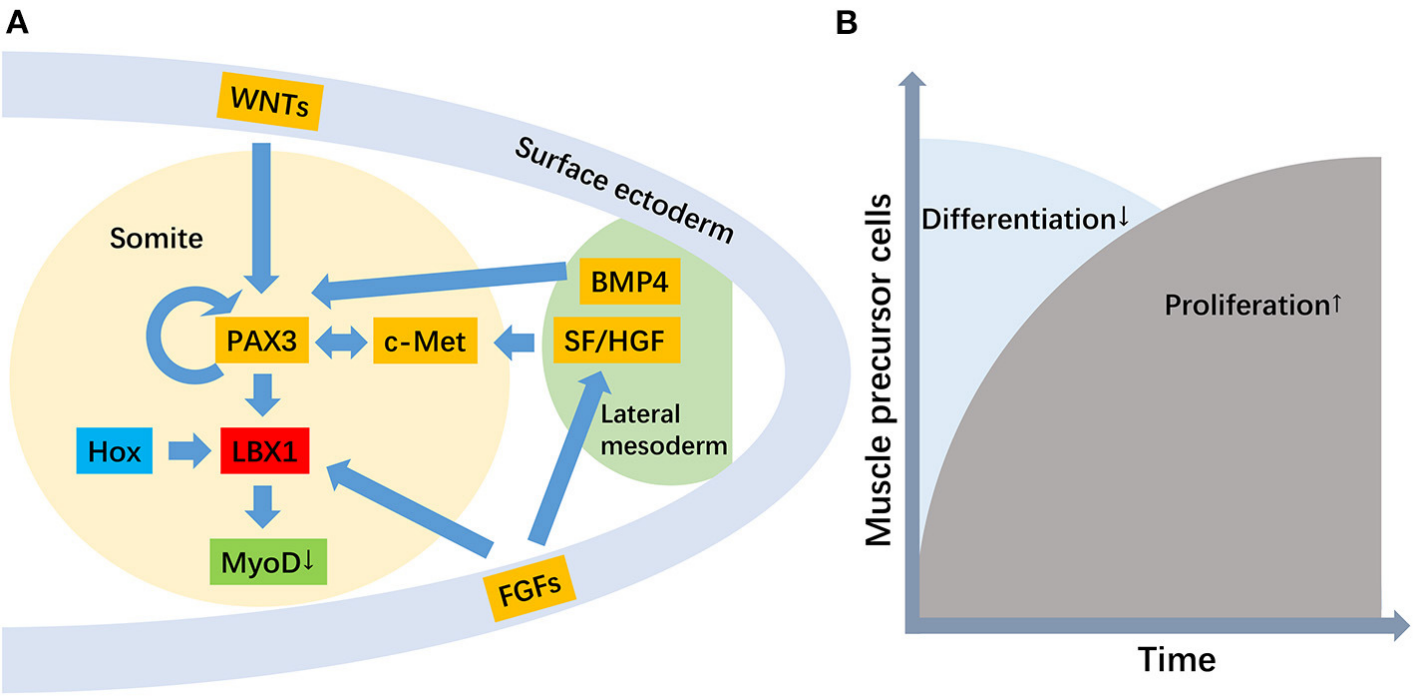
The role of the LBX1 gene in the migration of muscle precursor cells. [**(A)** The genetic hierarchy for the development of migratory muscle precursor cells, where the primary function of LBX1 is to repress MyoD. **(B)** The role of the LBX1 gene is the promotion of proliferation but the repression of the differentiation of migratory muscle precursor cells].

The LBX1 gene plays an essential role in regulating muscle precursor cell migration and maintaining its migratory potential (Figure 1) (Brohmann et al., 2000). Previous transcript analyses and immunofluorescence staining confirmed the upregulation and location of LBX1 in muscle precursor cells (Schmitteckert et al., 2011). Migratory muscle precursor cells could remain undifferentiated during migration, and LBX1+ cells commence differentiation into skeletal muscle cells after arrival in the limb (Tani-Matsuhana et al., 2018). In LBX1 knockout mice, the limb muscles were specifically lacking, whereas other skeletal muscles developed normally (Schäfer and Braun, 1999). Further evidence suggested that LBX1 regulated responsiveness to the lateral, but not the ventral, migration of hypaxial muscle precursors (Gross et al., 2000), potentially through ERK and FGF8 signaling to control lateral migration (Masselink et al., 2017).

A secondary role for LBX1 in promoting the proliferation of muscle precursor cells has also been proposed (Brohmann et al., 2000; Uchiyama et al., 2000). In Drosophila and their vertebrate counterparts, the LBX1 gene displayed a restricted expression pattern in a subset of muscle precursor cells and was implicated in the diversification of muscle cell fates (Bidet et al., 2003) The critical function of LBX1 is to expand the muscle precursor cell pool to respond to environmental stimulation (Mennerich and Braun, 2001). LBX1 is expressed in Xenopus myoblasts, and its function in hypaxial muscle development is to repress myoD, thereby allowing myoblasts to proliferate before terminal differentiation (Martin and Harland, 2006). Therefore, migration defects with LBX1 loss of function may be secondary to less efficient and reduced numbers of muscle precursor cells reaching their distal target. In addition, the expression of the LBX1 gene was also found in activated but not quiescent satellite cells, which suggested that LBX1 plays important roles in satellite cells (Watanabe et al., 2007).

The PAX3–LBX1 myogenic pathway as a whole appears to be conserved in elasmobranchs in a manner consistent with the patterning of limb muscle formation (Okamoto et al., 2017). In the absence of PAX3 in mice, LBX1 and c-Met expression in the somite was severely compromised, which suggested that PAX3 was necessary for LBX1 expression in the lateral tips of somites (Mennerich et al., 1998; Brohmann et al., 2000). Recently, PAX3 was proposed to act as a core regulator of the lateral migration of myoblasts, which has been hypothesized to be controlled by LBX1 (Masselink et al., 2017). A previous study identified rs13398147 near PAX3 as an AIS susceptibility locus in a Chinese population (Zhu et al., 2015), and PAX3 might have a functional role in the pathogenesis of AIS by regulating the development of paravertebral muscles (Qin et al., 2020). Significantly asymmetric bilateral expression of LBX1 and PAX3, two previously reported susceptible genes of AIS, was found in the paraspinal muscles of AIS patients. It is noteworthy that both genes were involved downstream of the Wnt/beta-catenin pathway (Zhu et al., 2017; Xu et al., 2020).

Paraspinal muscles play an important role in spinal stability, and muscle-based mechanisms are a possible etiology for AIS. Several studies have reported the asymmetry of paraspinal muscles between convex and concave side in patients with AIS. Paraspinal muscle thickness of mild AIS was measured using ultrasound imaging, and significantly greater muscle thickness was found on the concave side at apical region (Zapata et al., 2015). Muscle fiber redistribution was reported in AIS, and decreased type I fiber was found on the concave side (Stetkarova et al., 2016). Consistently, electrophysiological activity and muscle energy consumption were also significantly decreased on the convexity (Newton Ede and Jones, 2016; Stetkarova et al., 2016; Federau et al., 2020). Recently, the potential role of the LBX1 gene in the propagation of AIS through paraspinal muscles was investigated, and remarkably asymmetric expression of mRNA and protein was found in the paraspinal muscles at the apical region of AIS patients (Xu et al., 2020). Therefore, the LBX1 gene might be involved in asymmetric muscle formation by regulating the proliferation and differentiation of muscle precursor cells. In addition, the potential role of the LBX1 gene in other tissues, such as interneurons, cannot be ignored.

## The Function of the LBX1 Gene in Interneuron Development

LBX1 masks a subset of interneurons in the spinal cord, and it is known to play a role in neuronal determination processes (Schubert et al., 2001; Schmitteckert et al., 2011). The expression of LBX1 distinguishes two major neuronal classes generated in the dorsal spinal cord. LBX1+ and LBX1- neurons settle in the superficial and deep dorsal horn, respectively. In LBX1-mutant mice, the morphology and neuronal circuitry of the dorsal horn are aberrant due to the dysfunction of LBX1+ neurons (Muller et al., 2002). In LBX1-knockout mice, the presumptive GABAergic neurons were transformed into glutamatergic cells, and LBX1 was proposed to determine neuron fate in the dorsal spinal cord at early embryonic stages (Kruger et al., 2002; Cheng et al., 2005; Nadadhur et al., 2018). LBX1 is required for the expression of GlyT2, NPY, and N/OFQ in dorsal spinal neurons, which was supposed to play a role in promoting GABAergic neuron fate (Huang et al., 2008). Similar to the PAX3–LBX1 myogenic pathway involved in muscle development, LBX1 and PAX3 might act together synergistically to control the generation of neuronal subtypes (Kruger et al., 2002).

LBX1 acts as a selector gene in the fate determination of somatosensory relay neurons. The somatosensory information was transferred in a large number of distinct sensory interneurons that organized in specific laminae within the dorsal spinal horn (John et al., 2005). In mice lacking LBX1, cell types that arise in the ventral alar plate acquire more dorsal identities. This results in the loss of dorsal horn association interneurons, excess production of commissural neurons, and disrupted sensory afferent innervation of the dorsal horn (Gross et al., 2002). In LBX1-mutant mice, viscerosensory relay neurons are specified at the expense of somatosensory relay neurons, and LBX1 is essential to specify dBLb neurons that generate somatosensory relay neurons (Sieber et al., 2007).

The upright posture is made possible by proprioceptive system based on cortical, subcortical, and medullary integration of multisensorial information, especially somesthetic input (Munoz-Rubke et al., 2017; Sim et al., 2018). Proprioceptive system provides direct information on position and movements of body segments (Blecher et al., 2018). The gait parameters of AIS patients were investigated, and somatosensory dysfunction in AIS patients showed to have an impact on dynamic balance control (Lao et al., 2008). In addition, more serious disturbance of dynamic proprioceptive system was found in AIS, which suggested an immaturity of the central nervous system, especially parietal cortex, with poor integration of dynamic proprioceptive afference (Le Berre et al., 2017). Therefore, sensory processing of postural stability in AIS patients could be an important analytical factor.

Although the dysfunction of proprioception in AIS patients has been widely recognized in clinical studies (Yekutiel et al., 1981; Le Berre et al., 2017; Sim et al., 2018), the direct evidence linking impaired proprioception with scoliosis is still absence. Surprisingly, peripubertal scoliosis was successfully developed without vertebral dysplasia and muscle asymmetry in null-mutant mice for RUNX3, which lack TrkC neurons connecting proprioceptive mechanoreceptors and the spinal cord (Blecher et al., 2017). The resemblance of animal models to the human condition of AIS offers a clue as to its etiology and expands the scope of the proprioceptive function in musculoskeletal development (Blecher et al., 2018). Proprioceptive inputs alter with balance tasks of varying difficulty, with the spinal reflex pathway being inhibited in favor of sensory input to the cortex as balance becomes increasingly challenged (Le Berre et al., 2017). The rapid progression of AIS is coincides with the postnatal maturation of muscle mechanosensors, as well as a substantial increase in muscle mass. Peripubertal scoliosis might therefore result from increasing mechanical burden placed on a proprioceptive-deficient spine (Blecher et al., 2017; Assaraf et al., 2020). Abnormal central respiratory rhythmogenesis was found in LBX1-deficient humans, and the LBX1 mutation selectively interfered with its ability to cooperate with PHOX2B and thus impaired the development of a small subpopulation of neurons essential for respiratory control (Pagliardini et al., 2008; Hernandez-Miranda et al., 2018). This unusual gene-gene interaction could also provide insight into the functional study of the LBX1 gene in AIS pathogenesis.

## Perspectives and Conclusions

Despite strong evidence that it is the most promising candidate gene, the direct or indirect connection between susceptibility loci and hypothetical disease-risk genes remains poorly understood. The genomic architecture around LBX1 on chromosome 10q24.31 reveals a highly conserved gene with extensive regulatory mechanisms. A chromosome conformation capture assay revealed that the genome region with the most significantly associated SNP (rs11190870) physically interacted with the promoter region of LBX1, and the risk allele showed higher transcriptional activity in HEK 293T cells (Guo et al., 2016). These results suggest that rs11190870 confers AIS susceptibility by upregulating LBX1 transcription. In chicken embryos and explant cultures, overexpression of LBX1 showed in a strong activation of myogenic markers including MHC in somites and limbs, but not in other ectopic locations (Mennerich and Braun, 2001). However, upregulating of LBX1 in Xenopus lead to enlarged somites due to an increase in cell proliferation, but a lack of differentiated muscle (Martin and Harland, 2006). Similarly, forced expression of LBX1 in C2C12 myoblast cells resulted in severe depression of myogenic differentiation and incomplete myotube formation (Watanabe et al., 2007). The AIS presenting as low lean mass of back muscles (Cheng et al., 2015). Whether the risk allele of rs11190870 could suppress muscle formation through upregulating the LBX1 is still undetermined.

**Figure 2:**
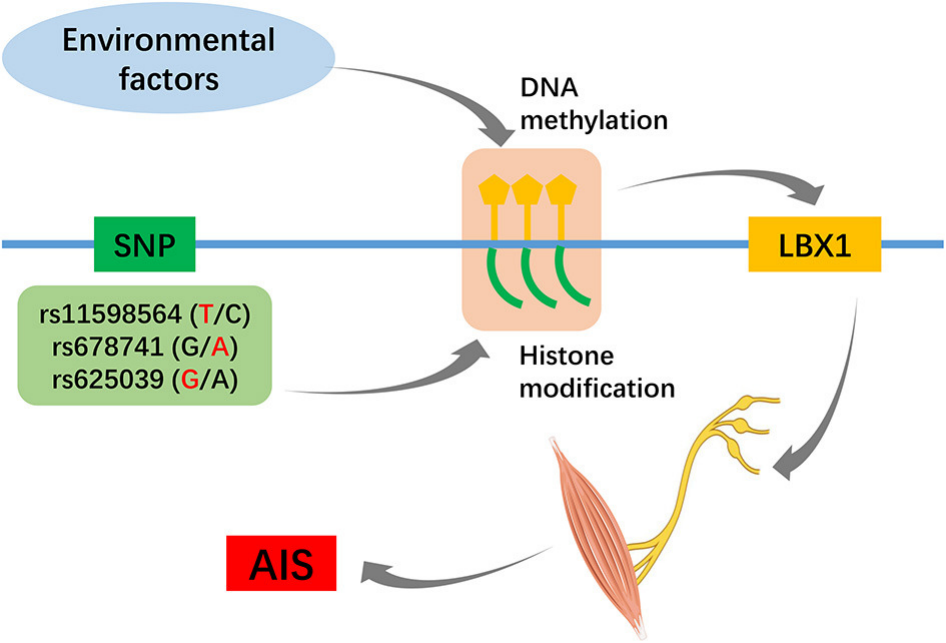
Hypothesis of susceptibility loci and epigenetic factors jointly regulate the LBX1 gene.

One hypothesis to explain the pathogenesis of AIS is that it is the consequence of crosstalk between multiple genes and environmental factors, and epigenetic analyses may complement genetic studies (Cheng et al., 2015; Ogura et al., 2018). Of particular interest is the head-to-head orientation of LBX1 with its antisense counterpart, LBX1-AS1, together with the very extensive CpG islands. Data from ENCODE showed that rs678741 is located in a strong enhancer region marked by peaks of several active histone methylation modifications (Zhu et al., 2015). Meng et al. (2018) found that decreased methylation at cg01374129 was associated with AIS curve progression, and hypomethylation at this site may influence adolescent spinal growth through altered HAS2 expression. Many researchers have realized the importance of epigenetics to account for the “missing heritability” in AIS GWAS, and increased methylation of the PCDH10 and COMP promoters was found in AIS patients relative to healthy controls (Mao et al., 2018; Shi et al., 2020). Further integrated genome-wide and epigenome-wide association studies (Figure 2) are necessary to reach a better understanding of the potential role of the LBX1 gene in AIS.

Although LBX1 is the most promising candidate gene in AIS susceptibility, which has been successfully replicated in multi-ethnic populations, GWASs just indicate statistically susceptible locations on the genome, not disease-causing sequence variations. The ability to replicate the phenotype of AIS using animal models is an urgent problem to be solved. Functional studies related to AIS susceptibility genes have been performed. Shorter body lengths and delayed ossification of the vertebrae were found in GPR126-knockdown zebrafish (Kou et al., 2013), mild spinal curvature caused by vertebral malformations was found in SLC39A8-knockout zebrafish (Haller et al., 2018), and body axis deformation was present in zebrafish with induced LBX1B overexpression in the embryonic period (Guo et al., 2016). However, these studies did not successfully replicate the human condition of AIS for pubertal scoliosis without vertebral dysplasia. Even worse, the overexpression or knockout of a gene has a much greater effect than the common variant near the candidate gene. Excitingly, the phenotype of spine malalignment without vertebral abnormalities was presented in PIEZO2 cKO mice, which indicated the central role of proprioceptive neurons in maintaining spinal stability (Assaraf et al., 2020). Therefore, the conversion of information on statistically susceptible loci near LBX1 to biological implications using animal models is of great importance to clarify the pathogenesis of AIS.

In conclusion, the LBX1 gene is the most promising candidate gene in AIS susceptibility due to its position and possible functions in muscle progenitor cell migration and neuronal determination processes. The causality between susceptibility loci related to the LBX1 gene and the pathogenesis of AIS deserves to be explored with further integrated genome-wide and epigenome-wide association studies.

## Author Contributions

ML and SH designed the study, ML and YZ collected data. ML and SH drafted and edited the manuscript, SH and YS supervised and edited. All authors approved the final manuscript.

## Conflict of Interest

The authors declare that the research was conducted in the absence of any commercial or financial relationships that could be construed as a potential conflict of interest.

## References

[B1] Agabegi S. S. Kazemi N. Sturm P. F. Mehlman C. T. (2015). Natural history of adolescent idiopathic scoliosis in skeletally mature patients: a critical review. J. Am. Acad. Orthop. Surg. 23, 714–723. 10.5435/JAAOS-D-14-00037 26510624

[B2] Assaraf E. Blecher R. Heinemann-Yerushalmi L. Krief S. Carmel Vinestock R. Biton I. E.. (2020). Piezo2 expressed in proprioceptive neurons is essential for skeletal integrity. Nat. Commun. 11:3168. 10.1038/s41467-020-16971-6 32576830 PMC7311488

[B3] Bidet Y. Jagla T. Da Ponte J. P. Dastugue B. Jagla K. (2003). Modifiers of muscle and heart cell fate specification identified by gain-of-function screen in Drosophila. Mech. Dev. 120, 991–1007. 10.1016/S0925-4773(03)00182-5 14550529

[B4] Blecher R. Heinemann-Yerushalmi L. Assaraf E. Konstantin N. Chapman J. R. Cope T. C.. (2018). New functions for the proprioceptive system in skeletal biology. Philos. Trans. R. Soc. London B Biol. Sci. 373:20170327. 10.1098/rstb.2017.0327 30249776 PMC6158198

[B5] Blecher R. Krief S. Galili T. Biton I. E. Stern T. Assaraf E.. (2017). The proprioceptive system masterminds spinal alignment: insight into the mechanism of scoliosis. Dev. Cell 42, 388–399.e3. 10.1016/j.devcel.2017.07.022 28829946

[B6] Brohmann H. Jagla K. Birchmeier C. (2000). The role of Lbx1 in migration of muscle precursor cells. Development 127, 437–445. 10603359 10.1242/dev.127.2.437

[B7] Cao Y. Min J. Zhang Q. Li H. Li H. (2016). Associations of LBX1 gene and adolescent idiopathic scoliosis susceptibility: a meta-analysis based on 34,626 subjects. BMC Musculoskelet. Disord. 17:309. 10.1186/s12891-016-1139-z 27450593 PMC4957912

[B8] Chen S. Zhao L. Roffey D. M. Phan P. Wai E. K. (2014). Association of rs11190870 near LBX1 with adolescent idiopathic scoliosis in East Asians: a systematic review and meta-analysis. Spine J. 14, 2968–2975. 10.1016/j.spinee.2014.05.019 24878781

[B9] Cheng J. C. Castelein R. M. Chu W. C. Danielsson A. J. Dobbs M. B. Grivas T. B. (2015). Adolescent idiopathic scoliosis. Nat. Rev. Dis. Primers 1:15030 10.1038/nrdp.2015.68 27188385

[B10] Cheng L. Samad O. A. Xu Y. Mizuguchi R. Luo P. Shirasawa S.. (2005). Lbx1 and Tlx3 are opposing switches in determining GABAergic versus glutamatergic transmitter phenotypes. Nat. Neurosci. 8, 1510–1515. 10.1038/nn1569 16234809

[B11] Chettier R. Nelson L. Ogilvie J. W. Albertsen H. M. Ward K. (2015). Haplotypes at LBX1 have distinct inheritance patterns with opposite effects in adolescent idiopathic scoliosis. PLoS ONE 10:e0117708. 10.1371/journal.pone.0117708 25675428 PMC4326419

[B12] Fan Y. H. Song Y. Q. Chan D. Takahashi Y. Ikegawa S. Matsumoto M.. (2012). SNP rs11190870 near LBX1 is associated with adolescent idiopathic scoliosis in southern Chinese. J. Hum. Genet. 57, 244–246. 10.1038/jhg.2012.11 22301463

[B13] Federau C. Kroismayr D. Dyer L. Farshad M. Pfirrmann C. (2020). Demonstration of asymmetric muscle perfusion of the back after exercise in patients with adolescent idiopathic scoliosis using intravoxel incoherent motion (IVIM) MRI. NMR Biomed. 33:e4194. 10.1002/nbm.4194 31815323

[B14] Gao W. Peng Y. Liang G. Liang A. Ye W. Zhang L.. (2013). Association between common variants near LBX1 and adolescent idiopathic scoliosis replicated in the Chinese Han population. PLoS ONE 8:e53234. 10.1371/journal.pone.0053234 23308168 PMC3537668

[B15] Grauers A. Wang J. Einarsdottir E. Simony A. Danielsson A. Akesson K.. (2015). Candidate gene analysis and exome sequencing confirm LBX1 as a susceptibility gene for idiopathic scoliosis. Spine J. 15, 2239–2246. 10.1016/j.spinee.2015.05.013 25987191

[B16] Gross M. K. Dottori M. Goulding M. (2002). Lbx1 specifies somatosensory association interneurons in the dorsal spinal cord. Neuron 34, 535–549. 10.1016/S0896-6273(02)00690-6 12062038

[B17] Gross M. K. Moran-Rivard L. Velasquez T. Nakatsu M. N. Jagla K. Goulding M. (2000). Lbx1 is required for muscle precursor migration along a lateral pathway into the limb. Development 127, 413–424. 10603357 10.1242/dev.127.2.413

[B18] Guo L. Yamashita H. Kou I. Takimoto A. Meguro-Horike M. Horike S.. (2016). Functional investigation of a non-coding variant associated with adolescent idiopathic scoliosis in zebrafish: elevated expression of the ladybird homeobox gene causes body axis deformation. PLoS Genet. 12:e1005802. 10.1371/journal.pgen.1005802 26820155 PMC4731154

[B19] Haller G. McCall K. Jenkitkasemwong S. Sadler B. Antunes L. Nikolov M.. (2018). A missense variant in SLC39A8 is associated with severe idiopathic scoliosis. Nat. Commun. 9:4171. 10.1038/s41467-018-06705-0 30301978 PMC6177404

[B20] Hernandez-Miranda L. R. Ibrahim D. M. Ruffault P. L. Larrosa M. Balueva K. Muller T.. (2018). Mutation in LBX1/Lbx1 precludes transcription factor cooperativity and causes congenital hypoventilation in humans and mice. Proc. Natl. Acad. Sci. U.S.A. 115, 13021–13026. 10.1073/pnas.1813520115 30487221 PMC6304989

[B21] Huang M. Huang T. Xiang Y. Xie Z. Chen Y. Yan R.. (2008). Ptf1a, Lbx1 and Pax2 coordinate glycinergic and peptidergic transmitter phenotypes in dorsal spinal inhibitory neurons. Dev. Biol. 322, 394–405. 10.1016/j.ydbio.2008.06.031 18634777

[B22] Jagla K. Dolle P. Mattei M. G. Jagla T. Schuhbaur B. Dretzen G.. (1995). Mouse Lbx1 and human LBX1 define a novel mammalian homeobox gene family related to the Drosophila lady bird genes. Mech. Dev. 53, 345–356. 10.1016/0925-4773(95)00450-5 8645601

[B23] Jiang H. Qiu X. Dai J. Yan H. Zhu Z. Qian B.. (2013). Association of rs11190870 near LBX1 with adolescent idiopathic scoliosis susceptibility in a Han Chinese population. Eur. Spine J. 22, 282–286. 10.1007/s00586-012-2532-4 23096252 PMC3555620

[B24] Jiang H. Yang Q. Liu Y. Guan Y. Zhan X. Xiao Z.. (2019). Association between ladybird homeobox 1 gene polymorphisms and adolescent idiopathic scoliosis: a MOOSE-compliant meta-analysis. Medicine 98:e16314. 10.1097/MD.0000000000016314 31277174 PMC6635165

[B25] John A. Wildner H. Britsch S. (2005). The homeodomain transcription factor Gbx1 identifies a subpopulation of late-born GABAergic interneurons in the developing dorsal spinal cord. Dev. Dyn. 234, 767–771. 10.1002/dvdy.20568 16193514

[B26] Kou I. Otomo N. Takeda K. Momozawa Y. Lu H. F. Kubo M.. (2019). Genome-wide association study identifies 14 previously unreported susceptibility loci for adolescent idiopathic scoliosis in Japanese. Nature Commun. 10:3685. 10.1038/s41467-019-11596-w 31417091 PMC6695451

[B27] Kou I. Takahashi Y. Johnson T. A. Takahashi A. Guo L. Dai J.. (2013). Genetic variants in GPR126 are associated with adolescent idiopathic scoliosis. Nat. Genet. 45, 676–679. 10.1038/ng.2639 23666238

[B28] Kruger M. Schafer K. Braun T. (2002). The homeobox containing gene Lbx1 is required for correct dorsal-ventral patterning of the neural tube. J. Neurochem. 82, 774–782. 10.1046/j.1471-4159.2002.01078.x 12358782

[B29] Lao M. L. Chow D. H. Guo X. Cheng J. C. Holmes A. D. (2008). Impaired dynamic balance control in adolescents with idiopathic scoliosis and abnormal somatosensory evoked potentials. J. Pediatr. Orthop. 28, 846–849. 10.1097/BPO.0b013e31818e1bc9 19034176

[B30] Le Berre M. Guyot M. A. Agnani O. Bourdeauducq I. Versyp M. C. Donze C.. (2017). Clinical balance tests, proprioceptive system and adolescent idiopathic scoliosis. Eur. Spine J. 26, 1638–1644. 10.1007/s00586-016-4802-z 27844226

[B31] Liang J. Xing D. Li Z. Chua S. Li S. (2014). Association between rs11190870 polymorphism Near LBX1 and susceptibility to adolescent idiopathic scoliosis in east asian population: a genetic meta-analysis. Spine 39, 862–869. 10.1097/BRS.0000000000000303 24583738

[B32] Liu S. Wu N. Zuo Y. Zhou Y. Liu J. Liu Z.. (2017). Genetic polymorphism of LBX1 is associated with adolescent idiopathic scoliosis in Northern Chinese Han population. Spine 42, 1125–1129. 10.1097/BRS.0000000000002111 28187071

[B33] Londono D. Kou I. Johnson T. A. Sharma S. Ogura Y. Tsunoda T.. (2014). A meta-analysis identifies adolescent idiopathic scoliosis association with LBX1 locus in multiple ethnic groups. J. Med. Genet. 51, 401–406. 10.1136/jmedgenet-2013-102067 24721834

[B34] Lonstein J. E. (2006). Scoliosis: surgical versus nonsurgical treatment. Clin. Orthop. Relat. Res. 443:248–259. 10.1097/01.blo.0000198725.54891.73 16462448

[B35] Man G. C. Tang N. L. Chan T. F. Lam T. P. Li J. W. Ng B. K.. (2019). Replication study for the association of GWAS-associated loci with adolescent idiopathic scoliosis susceptibility and curve progression in a Chinese population. Spine 44, 464–471. 10.1097/BRS.0000000000002866 30234802

[B36] Mao S. H. Qian B. P. Shi B. Zhu Z. Z. Qiu Y. (2018). Quantitative evaluation of the relationship between COMP promoter methylation and the susceptibility and curve progression of adolescent idiopathic scoliosis. Eur. Spine J. 27, 272–277. 10.1007/s00586-017-5309-y 28951969

[B37] Martin B. L. Harland R. M. (2006). A novel role for lbx1 in xenopus hypaxial myogenesis. Development 133, 195–208. 10.1242/dev.02183 16339190

[B38] Masselink W. Masaki M. Sieiro D. Marcelle C. Currie P. D. (2017). Phosphorylation of Lbx1 controls lateral myoblast migration into the limb. Dev. Biol. 430, 302–309. 10.1016/j.ydbio.2017.08.025 28843494

[B39] Meng Y. Lin T. Liang S. Gao R. Jiang H. Shao W.. (2018). Value of DNA methylation in predicting curve progression in patients with adolescent idiopathic scoliosis. EBioMed. 36, 489–496. 10.1016/j.ebiom.2018.09.014 30241917 PMC6197569

[B40] Mennerich D. Braun T. (2001). Activation of myogenesis by the homeobox gene Lbx1 requires cell proliferation. EMBO J. 20, 7174–7183. 10.1093/emboj/20.24.7174 11742994 PMC125799

[B41] Mennerich D. Schafer K. Braun T. (1998). Pax-3 is necessary but not sufficient for lbx1 expression in myogenic precursor cells of the limb. Mech. Dev. 73, 147–158. 10.1016/S0925-4773(98)00046-X 9622616

[B42] Muller T. Brohmann H. Pierani A. Heppenstall P. A. Lewin G. R. Jessell T. M.. (2002). The homeodomain factor lbx1 distinguishes two major programs of neuronal differentiation in the dorsal spinal cord. Neuron 34, 551–562. 10.1016/S0896-6273(02)00689-X 12062039

[B43] Munoz-Rubke F. Mirdamadi J. L. Lynch A. K. Block H. J. (2017). Modality-specific changes in motor cortex excitability after visuo-proprioceptive realignment. J. Cogn. Neurosci. 29, 2054–2067. 10.1162/jocn_a_01171 28777059

[B44] Nada D. Julien C. Samuels M. E. Moreau A. (2018). A replication study for association of LBX1 locus with adolescent idiopathic scoliosis in French-Canadian population. Spine 43, 172–178. 10.1097/BRS.0000000000002280 28604496

[B45] Nadadhur A. G. Leferink P. S. Holmes D. Hinz L. Cornelissen-Steijger P. Gasparotto L.. (2018). Patterning factors during neural progenitor induction determine regional identity and differentiation potential *in vitro*. Stem Cell Res. 32, 25–34. 10.1016/j.scr.2018.08.017 30172094

[B46] Newton Ede M. M. Jones S. W. (2016). Adolescent idiopathic scoliosis: evidence for intrinsic factors driving aetiology and progression. Int. Orthop. 40, 2075–2080. 10.1007/s00264-016-3132-4 26961194

[B47] Ogilvie J. (2010). Adolescent idiopathic scoliosis and genetic testing. Curr. Opin. Pediatr. 22, 67–70. 10.1097/MOP.0b013e32833419ac 19949338

[B48] Ogura Y. Matsumoto M. Ikegawa S. Watanabe K. (2018). Epigenetics for curve progression of adolescent idiopathic scoliosis. EBioMed. 37:36–37. 10.1016/j.ebiom.2018.10.015 30316863 PMC6284410

[B49] Ogura Y. Takahashi Y. Kou I. Nakajima M. Kono K. Kawakami N.. (2013). A replication study for association of 53 single nucleotide polymorphisms in a scoliosis prognostic test with progression of adolescent idiopathic scoliosis in Japanese. Spine 38, 1375–1379. 10.1097/BRS.0b013e3182947d21 23591653

[B50] Okamoto E. Kusakabe R. Kuraku S. Hyodo S. Robert-Moreno A. Onimaru K. (2017). Migratory appendicular muscles precursor cells in the common ancestor to all vertebrates. Nat. Ecol. Evol. 1, 1731–1736. 10.1038/s41559-017-0330-4 28970537

[B51] Pagliardini S. Ren J. Gray P. A. Vandunk C. Gross M. Goulding M.. (2008). Central respiratory rhythmogenesis is abnormal in lbx1- deficient mice. J. Neurosci. 28, 11030–11041. 10.1523/JNEUROSCI.1648-08.2008 18945911 PMC6671375

[B52] Qin X. He Z. Yin R. Qiu Y. Zhu Z. (2020). Abnormal paravertebral muscles development is associated with abnormal expression of PAX3 in adolescent idiopathic scoliosis. Eur. Spine J. 29, 737–743. 10.1007/s00586-019-06217-5 31832874

[B53] Roye B. D. Wright M. L. Matsumoto H. Yorgova P. McCalla D. Hyman J. E.. (2015). An independent evaluation of the validity of a DNA-based prognostic test for adolescent idiopathic scoliosis. J. Bone Joint Surg. Am. 97, 1994–1998. 10.2106/JBJS.O.00217 26677232

[B54] Schäfer K. Braun T. (1999). Early specification of limb muscle precursor cells by the homeobox gene Lbx1h. Nat. Genet. 23, 213–216. 10.1038/13843 10508520

[B55] Schmitteckert S. Ziegler C. Kartes L. Rolletschek A. (2011). Transcription factor lbx1 expression in mouse embryonic stem cell-derived phenotypes. Stem Cells Int 2011:130970. 10.4061/2011/130970 21941564 PMC3175398

[B56] Schubert F. R. Dietrich S. Mootoosamy R. C. Chapman S. C. Lumsden A. (2001). Lbx1 marks a subset of interneurons in chick hindbrain and spinal cord. Mech. Dev. 101, 181–185. 10.1016/S0925-4773(00)00537-2 11231071

[B57] Shi B. Mao S. Xu L. Li Y. Sun X. Liu Z.. (2020). Quantitation analysis of PCDH10 methylation in adolescent idiopathic scoliosis using pyrosequencing study. Spine 45, E373–E378. 10.1097/BRS.0000000000003292 31651684

[B58] Sieber M. A. Storm R. Martinez-de-la-Torre M. Muller T. Wende H. Reuter K.. (2007). Lbx1 acts as a selector gene in the fate determination of somatosensory and viscerosensory relay neurons in the hindbrain. J. Neurosci. 27, 4902–4909. 10.1523/JNEUROSCI.0717-07.2007 17475798 PMC6672097

[B59] Sim T. Yoo H. Lee D. Suh S. W. Yang J. H. Kim H.. (2018). Analysis of sensory system aspects of postural stability during quiet standing in adolescent idiopathic scoliosis patients. J. Neuroeng. Rehabil. 15:54. 10.1186/s12984-018-0395-6 29929530 PMC6013903

[B60] Simony A. Carreon L. Y. Hjmark K. Kyvik K. O. Andersen M. (2016). Concordance rates of adolescent idiopathic scoliosis in a danish twin population. Spine 41, 1503–1507. 10.1097/BRS.0000000000001681 27163371

[B61] Stetkarova I. Zamecnik J. Bocek V. Vasko P. Brabec K. Krbec M. (2016). Electrophysiological and histological changes of paraspinal muscles in adolescent idiopathic scoliosis. Eur. Spine J. 25, 3146–3153. 10.1007/s00586-016-4628-8 27246349

[B62] Takahashi Y. Inaba I. Kono K. Kawakami N. Uno K. Ito M. (2015). rs11190870 is not associated with severity of adolescent idiopathic scoliosis in Japanese. Scoliosis 10:O3 10.1186/1748-7161-10-S1-O3

[B63] Takahashi Y. Kou I. Ogura Y. Miyake A. Takeda K. Nakajima M.. (2018). A replication study for the association of rs11190870 with curve severity in adolescent idiopathic scoliosis in Japanese. Spine 43, 688–692. 10.1097/BRS.0000000000002413 28902104

[B64] Takahashi Y. Kou I. Takahashi A. Johnson T. A. Kono K. Kawakami N.. (2011). A genome-wide association study identifies common variants near LBX1 associated with adolescent idiopathic scoliosis. Nat. Genet. 43, 1237–1240. 10.1038/ng.974 22019779

[B65] Tang Q. L. Julien C. Eveleigh R. Bourque G. Franco A. Labelle H.. (2015). A replication study for association of 53 single nucleotide polymorphisms in ScoliScore test with adolescent idiopathic scoliosis in French-Canadian population. Spine 40, 537–543. 10.1097/BRS.0000000000000807 25646748

[B66] Tani-Matsuhana S. Kusakabe R. Inoue K. (2018). Developmental mechanisms of migratory muscle precursors in medaka pectoral fin formation. Dev. Genes Evol. 228, 189–196. 10.1007/s00427-018-0616-9 30008036

[B67] Uchiyama K. Ishikawa A. Hanaoka K. (2000). Expression of lbx1 involved in the hypaxial musculature formation of the mouse embryo. J. Experimental Zool. 286, 270–279. 10.1002/(SICI)1097-010X(20000215)286:3<270::AID-JEZ6>3.0.CO;2-P 10653966

[B68] Ward K. Ogilvie J. W. Singleton M. V. Chettier R. Engler G. Nelson L. M. (2010). Validation of DNA-based prognostic testing to predict spinal curve progression in adolescent idiopathic scoliosis. Spine 35, E1455–E1464. 10.1097/BRS.0b013e3181ed2de1 21102273

[B69] Watanabe S. Kondo S. Hayasaka M. Hanaoka K. (2007). Functional analysis of homeodomain-containing transcription factor Lbx1 in satellite cells of mouse skeletal muscle. J. Cell Sci. 120(Pt. 23), 4178–4187. 10.1242/jcs.011668 18003701

[B70] Weinstein S. L. Dolan L. A. Wright J. G. Dobbs M. B. (2013). Effects of bracing in adolescents with idiopathic scoliosis. N. Engl. J. Med. 369, 1512–1521. 10.1056/NEJMoa1307337 24047455 PMC3913566

[B71] Xu L. Dai Z. Xia C. Wu Z. Feng Z. Sun X. (2020). Asymmetric expression of Wnt/B-catenin pathway in AIS: primary or secondary to the curve? Spine 45, E677–E683. 10.1097/BRS.0000000000003409 32044811

[B72] Xu L. Qin X. Sun W. Qiao J. Qiu Y. Zhu Z. (2016). Replication of association between 53 single-nucleotide polymorphisms in a DNA-based diagnostic test and AIS progression in Chinese Han population. Spine 41, 306–310. 10.1097/BRS.0000000000001203 26579958

[B73] Yee A. Song Y. Q. Chan D. Cheung K. M. (2014). Understanding the basis of genetic studies: adolescent idiopathic scoliosis as an example. Spine Deform. 2, 1–9. 10.1016/j.jspd.2013.09.003 27927437

[B74] Yekutiel M. Robin G. C. Yarom R. (1981). Proprioceptive function in children with adolescent idiopathic scoliosis. Spine 6, 560–566. 10.1097/00007632-198111000-00006 7336278

[B75] Zapata K. A. Wang-Price S. S. Sucato D. J. Dempsey-Robertson M. (2015). Ultrasonographic measurements of paraspinal muscle thickness in adolescent idiopathic scoliosis: a comparison and reliability study. Pediatr. Phys. Ther. 27, 119–125. 10.1097/PEP.0000000000000131 25695194

[B76] Zhu Z. Tang N. L. Xu L. Qin X. Mao S. Song Y.. (2015). Genome-wide association study identifies new susceptibility loci for adolescent idiopathic scoliosis in Chinese girls. Nat. Commun. 6:8355. 10.1038/ncomms9355 26394188 PMC4595747

[B77] Zhu Z. Xu L. Leung-Sang Tang N. Qin X. Feng Z. Sun W.. (2017). Genome-wide association study identifies novel susceptible loci and highlights Wnt/beta-catenin pathway in the development of adolescent idiopathic scoliosis. Hum. Mol. Genet. 26, 1577–1583. 10.1093/hmg/ddx045 28334814

